# The Role of Pre-ablative Stimulated Thyroglobulin and Thyroglobulin/ Thyroid-Stimulating Hormone Ratio for Predicting Metastasis in Thyroid Cancer

**DOI:** 10.4274/mirt.galenos.2018.09825

**Published:** 2019-03-19

**Authors:** Fadime Demir, Fikri Selçuk Şimşek, Tansel Ansal Balcı

**Affiliations:** 1 Tokat Gaziosmanpaşa University Faculty of Medicine, Department of Nuclear Medicine, Tokat, Turkey; 2 Fırat University Faculty of Medicine, Department of Nuclear Medicine, Elazığ, Turkey

**Keywords:** Thyroid cancer, thyroglobulin, metastasis

## Abstract

**Objectives:**

In this study, we aimed to investigate the predictive value of pre-ablative stimulated thyroglobulin (Tg) and Tg/thyroid-stimulating hormone (TSH) to identify lymph node metastasis (LNM) or distant metastases (DM) prior to radioactive iodine (RAI) treatment.

**Methods:**

Patients without metastasis were included in group 1 (n=100), those with LNM were included in group 2 (n=83), and those with DM constituted group 3 (n=23). Tg and TSH values were measured approximately 4 hours prior to RAI ablation therapy.

**Results:**

There was a significant difference between group 3 and other groups (group 1 and group 2) in terms of Tg (p<0.001) and Tg/ TSH (p<0.001). For Tg level and Tg/TSH ratio, the areas under ROC were 0.990 [95% confidence interval (CI): 0.979-1] and 0.991 (95% CI: 0.981-1), respectively. The cut-off points for Tg and Tg/TSH were 102 ng/mL and 1.06, respectively.

**Conclusion:**

Our results suggest that Tg and Tg/TSH values can be used to predict DM. On the other hand, our study indicates that patients should be carefully evaluated for LNM even when Tg levels are low.

## Abstract (tr)

**Amaç:**

Bu çalışmada, radyoaktif iyot (RAİ) tedavisi öncesi lenf nodu metastazını veya uzak metastazları belirlemek için pre-ablatif stimüle tiroglobulin (Tg) ve Tg/tiroid-uyarıcı hormonun (Tg/TSH) prediktif değerini araştırmayı amaçladık.

**Yöntem:**

Grup 1’e (n=100) metastaz saptanmayan hastalar, grup 2’ye (n=83) lenf nodu metastazı olan hastalar ve grup 3’e (n=23) uzak metastazı olan hastalar dahil edildi. Tg ve TSH değerleri RAİ tedavisinden yaklaşık 4 saat önce ölçüldü

**Bulgular:**

Grup 3 ile diğer gruplar (grup 1 ve grup 2) arasında Tg (p<0,001) ve Tg/TSH (p<0,001) açısından anlamlı fark vardı. Tg seviyesi ve Tg/TSH oranı için ROC eğrisi altındaki alanlar sırasıyla 0,990 (%95 güven aralığı: 0,979-1) ve 0,991 (%95 güven aralığı: 0,981-1) idi. Tg ve Tg/ TSH için kesme noktaları sırasıyla 102 ng/mL ve 1,06 ng/mL idi.

**Sonuç:**

Çalışmamıza göre Tg ve Tg/TSH değerleri uzak metastaz için prediktif bir değer olarak kullanılabilir. Öte yandan, çalışmamız lenf nodu metastazının düşük Tg seviyelerinde bile dikkatli bir şekilde değerlendirilmesi gerektiğini düşündürmektedir.

## Introduction

Thyroid cancer is the most common endocrine malignancy worldwide, with a rapidly inceraseing incidence rate (1). Thyroid carcinomas are classified as differentiated or undifferentiated according to their histologic type. Differentiated thyroid carcinomas (DTC) account >90% of thyroid cancer. The standard treatments for DTC include total thyroidectomy (TT), radioactive iodine (RAI) ablation therapy (patients with a tumor >1 cm in size) and long-term thyroid stimulating hormone (TSH) suppression therapy (2). DTC has a relatively good prognosis with 10‑year survival rates of 92‑98%. Nevertheless, cervical lymph node metastases (LNM) develop in 53% and distant metastases (DM) in 10% of patients (3,4). The RAI dose to administer can be chosen either empirically (100-200 mCi) or by lesional or whole-body dosimetry if available, in order to limit the whole-body retention to 80 mCi at 48 hours and 200 cGy to the bone marrow (5). The most common method is empiric administration in which the radioiodine dose is based primarily on the extent of the tumor. The potential disadvantage of empiric dosing is that individual patients may be under- or over-dosed (6). Presence of LNM and DM are significant determinants for empirical dose planning. LNM or DM can be detected by using clinical evaluation, as well as surgical, radiological and diagnostic iodine-131 (I-131) whole-body scan (WBS) findings. However, it will be more appropriate if diagnostic tools are performed after a risk assessment or clinical suspicion. Thyroglobulin (Tg) is the specific marker of thyroid tissue. Tg levels significantly decrease after surgical removal of thyroid tissue, while Tg levels remain high in case of residual tissue or DM in thyroid cancer (7). Endogenous TSH can stimulate Tg release from the thyroid bed or metastatic tissue. Endogenous TSH induce Tg release from thyroid bed or metastatic tissue. This means that the Tg release is dependent by TSH (8). The aim of this study was to investigate the potential value of pre-ablative stimulated Tg and Tg/TSH to identify LNM or DM prior to RAI treatment.

## Materials and Methods

### Patients

Patients treated with RAI for thyroid cancer in Fırat University Hospital between 2012 and 2018 were reviewed in this retrospective analysis. One hundred patients without metastasis were included in group 1, eighty-three patients with lymph node metastasis were included in group 2 and 23 patients with DM were included in group 3. Metastasis was diagnosed by pathologic involvement in whole body RAI scan after treatment, with or without positive findings on other imaging modalities [computed tomography (CT), magnetic resonance (MR), and positron emission tomograph/CT]. Patients with positive anti-Tg antibodies (TgAb) were excluded from the study, since their Tg levels could have been affected. This retrospective analysis has been approved by the Fırat University Research Committee (06.09.2018/14-10).

### Radioiodine Therapy and Follow-up

Thyroid hormone replacement was withdrawn for 3-4 weeks prior to RAI treatment, and patients’ TSH levels were increased over 30 IU/mL if possible. Patients followed a low-iodine diet for 10 days before I-131 treatment. The doses of radioiodine were determined by performing post-op neck ultrasonography (USG) with or without MR, Tc-99m thyroid scan, along with Tg values. For radioiodine ablation, a dose of 3.7 GBq was administered to eliminate thyroid remnants. When lymph node metastases were detected, patients were treated with radioiodine at a dose of 5.55 GBq. If DM was detected, patients were treated with radioiodine at a dose of 7.4 GBq. I-131 WBS was performed 7-8 days after treatment of I-131.

### Tg and TSH Measurement

Tg and TSH were measured approximately 4 hours before RAI administration. Tg levels were determined by chemiluminescence immunoassay (IMMULITE^®^ 2000 XPi Immunoassay System, US/Wales, UK). Measuring ranges were 0.20 to 30000 ng/mL (with 1/100 dilution). TSH levels were determined by chemiluminescence immunoassay (ADVIA Centaur CP Immunoassay System/US) Measuring ranges were 0.010 to 150 µIU/L. TgAb were determined by chemiluminescence microparticle immunoassay (ARCHITECT i2000SR). Measuring ranges were 20 to 1000 IU/mL. Positivity for TgAb was accepted as more than 40 IU/mL, and patients with TgAb levels above 40 IU/mL were excluded from the study.

### Statistical Analysis

Continuous variables are reported as mean ± standard deviation or median values and ranges, while categorical variables are reported as absolute numbers. Between groups, differences were assessed with the Kruskal-Wallis test (and Mann-Whitney U pair-wise comparisons) or the chi‑square test (categorical variables). A p value less than 0.05 was considered as significant. Receiver-operating characteristic (ROC) curve analysis was used to define the best cut-off value for serum Tg in terms of showing the presence of metastases. For the established cut-off value, we calculated the sensitivity, specificity, and area under the curve (AUC). All analyses were performed with SPSS Software (version 20.0).

## Results

Of the 206 patients included in the study, 155 were female and 51 were male. The mean age was 45.88±13.59. Characteristics of study subjects are presented in Table 1.

**Figure 1:**
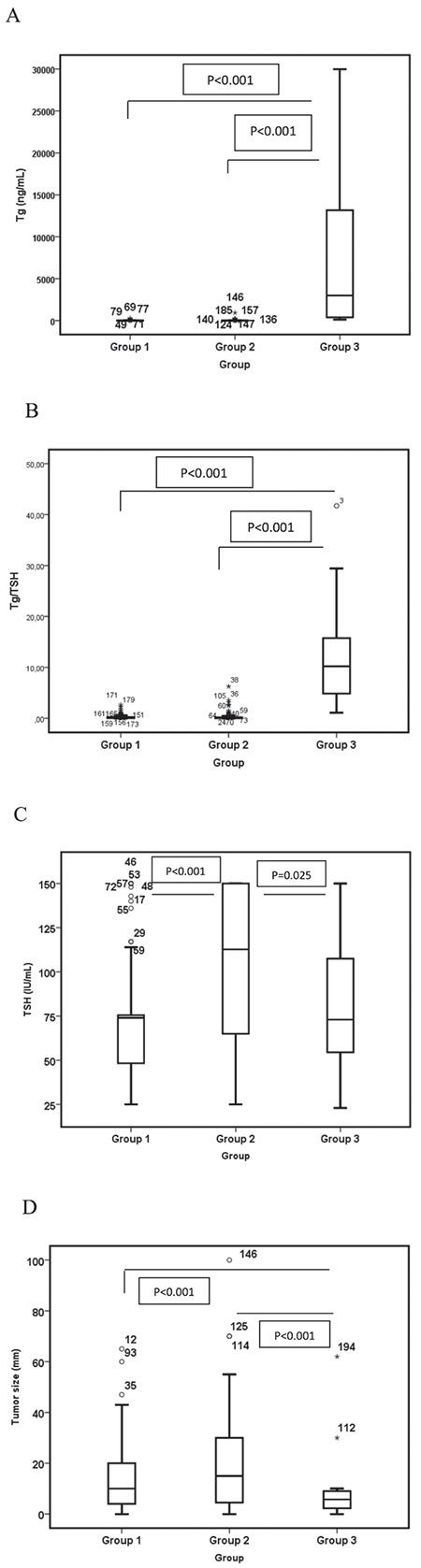
Association of characteristics between groups by Kruskal-Wallis test and Mann-Whitney U pair-wise comparisons: A) Comparison of groups in terms of thyroglobulin (Tg) values. B) Comparison of groups in terms of Tg/thyroid-stimulating hormone (TSH). C) Comparison of groups in terms of TSH. D) Comparison of groups in terms of tumor size

There was a significant difference between group 3 and other groups (group 1 and group 2) in terms of Tg (p<0.001) and Tg/TSH (p<0.001). In group 3, Tg and Tg/TSH were higher than the other groups. But there was no significant difference between group 1 and group 2 (Figure 1). There was also a significant difference in terms of gender (p<0.001) and age (p<0.001) between groups (Table 2). In group 3, the tumor size was significantly lower than group 1 and group 2 (p<0.001).

**Figure 2:**
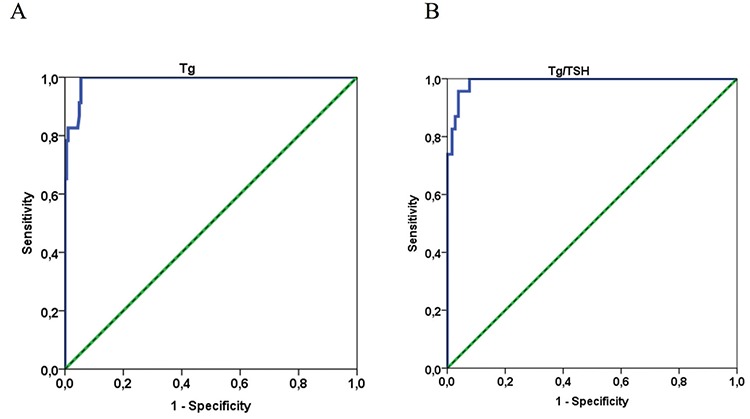
Receiving operator characteristic (ROC) curve for thyroglobulin (Tg) and Tg/thyroid-stimulating hormone (TSH) to detect distant metastatic differentiated thyroid carcinoma. A) ROC curve for Tg level. B) ROC curve for Tg/TSH ratio

The diagnostic accuracy of serum Tg and Tg/TSH was evaluated using ROC analysis. The ROC curve is illustrated in Figure 2. The areas under ROC for Tg level and Tg/TSH ratios were 0.990 [95% confidence interval (CI): 0.979-1] and 0.991 (95% CI: 0.981-1), respectively. The cut-off point was specified from the ROC curve using the optimal intersection of specificity and sensitivity. Based on the drawn ROC curve, the cut-off point for Tg was at 102 ng/mL (sensitivity; 100%, specificity; 94.5%) and for Tg/TSH was at 1.06 (sensitivity; 100%, specificity; 92.3%).

## Discussion

LNM is known as a risk factor for poor clinical outcome in thyroid carcinoma. Decreased survival and increased mortality rates have been demonstrated among patients with DTC with lymph node metastasis (9). 10-15% of patients with DTC present with or subsequently develop DM. In these patients, the 10-year disease-specific survival rate drops to 40% (10). Early detection and treatment have been found to have a substantial effect on the survival rate of patients with DTC (11). Detection of metastasis of DTC patients is important for better treatment planning. USG, chest radiography, CT, MR and diagnostic WBS are imaging modalities used for LNM and DM diagnosis. Nevertheless, sometimes it may not be visualized on these imaging techniques (11) and the metastasis can only be detected in WBS after treatment.

Tg is the specific marker of thyroid tissue. Tg levels significantly decrease after surgical removal of thyroid tissue, while Tg levels remain high in case of residual tissue or DM in thyroid cancer (7). Therefore Tg is a tumor marker for therapy monitoring and a significant parameter used in the follow-up of subjects with DTC. Excluding thyroid cell damage, two factors determine Tg concentration in most clinical situations. These factors are thyroid cell mass and activation of TSH receptors (12). TSH secretion induced by LT4 withdrawal increases the sensitivity of Tg measurement in terms of neoplastic tissue detection (13). Since TSH values of pre-ablative patients may be different, Tg values may also be affected accordingly. For this reason, in our study, we included Tg/TSH ratio in our study parameters in addition to Tg to investigate the predictive value for metastasis in patients with DTC.

According to the results of our study; there was a significant difference between the group without metastasis and with DM in terms of both Tg and Tg/TSH values. ROC analysis of Tg and Tg/TSH also showed good accuracy (0.990 and 0.991) as diagnostic markers for DM. In a similar study by Lin et al. (14), they reported that both Tg and Tg/TSH ratios could be considered predictors of DTC DM after TT prior to the first I-131 ablative therapy. Area under the ROC curve for Tg concentrations and Tg/TSH ratios were 0.913 and 0.916, respectively, in this study.

In a study which investigates the value of pre-ablation stimulated Tg in predicting DM of papillary thyroid cancer (15), it was reported that area under the ROC curve for Tg levels was 0.893 and the cut-off value of Tg was 52.75 µg/L with a sensitivity of 78.90% and specificity of 91.70%. In our study, we found that the areas under ROC for Tg level was 0.990, the cut-off point for Tg was at 102 ng/mL. We think that this difference in Tg cut-off may be related to the Tg measurement method.

In an analysis of Tg doubling time (Tg-DT), which is the time required to double the amount of Tg, Rössing et al. (16) have suggested that Tg-DT is not a single predictor of progressive disease but that it creates significant differences in the survival of patients with high tumor burden in patients with progressive DTC. They reported that there is a significant difference in survival rates patients with Tg levels greater than 100 ng/mL and with Tg levels lower than 100 ng/mL. This result suggested that one of the reasons for the difference in survival rate detected in their study might be DM. Zhao et al. (17) suggested that pre-ablative Tg levels may be affected by TSH and residual tissue after surgery, therefore, the difference between serial Tg measurements (at an average 8-day interval) could be a better marker of DM. The area under the ROC curve for ΔTg (ΔTg <0, ΔTg >0) and ΔTg/ΔTSH (ΔTg/ΔTSH <0, ΔTg/ΔTSH >0) parameters in their study was 0.907, 0.856 and 0.911, 0.905, respectively. Based on the drawn ROC curve, the cut-off point for DTg was at -6.55–3.90 ng/mL and for ΔTg/ΔTSH was at -0.40–0.41 ng/µIU. 

In our study, there was no significant difference between patients with lymph node metastasis and those without metastasis in terms of Tg and Tg/TSH values. This result suggests that these parameters could not be used to predict LNM. In the literature, the group of patients with metastasis are classified as those with combined lymph node and DM or with DM alone. To the best of our knowledge, there aren’t any studies comparing patients with and without lymph node metastases in terms of postoperative stimulated Tg values. Ronga et al. (18) reported that the mean Tg value was not significantly different between those with lymph node metastases and those with DM. In our study, both Tg and Tg/TSH values were significantly different between these two groups.

## Conclusion

In conclusion, our results suggest that preablative Tg and Tg/TSH values can be used to estimate DM. On the other hand, these values do not contribute significantly to the estimation of lymph node metastasis; therefore, we think that patients should be evaluated carefully for LNM even if their Tg levels are low.

## Figures and Tables

**Table 1:**
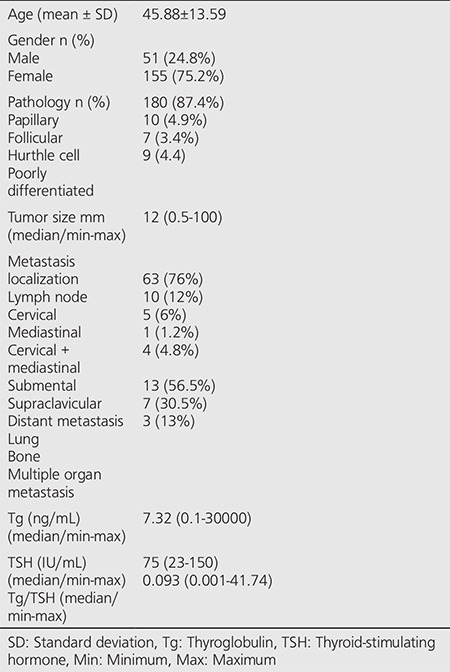
Characteristics of study subjects

**Table 2:**
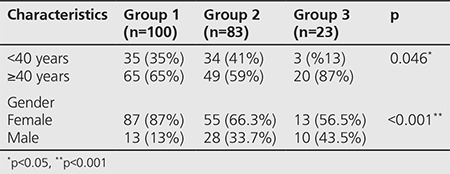
Comparison of characteristics between groups
